# Cell competition in primary and metastatic colorectal cancer

**DOI:** 10.1038/s41389-024-00530-5

**Published:** 2024-07-26

**Authors:** Merel Elise van Luyk, Ana Krotenberg Garcia, Maria Lamprou, Saskia Jacoba Elisabeth Suijkerbuijk

**Affiliations:** Division of Developmental Biology, Institute of Biodynamics and Biocomplexity, Department of Biology, Faculty of Science, Utrecht University, Utrecht, The Netherlands

**Keywords:** Colorectal cancer, Metastasis, Cell growth, Stem cells

## Abstract

Adult tissues set the scene for a continuous battle between cells, where a comparison of cellular fitness results in the elimination of weaker “loser” cells. This phenomenon, named cell competition, is beneficial for tissue integrity and homeostasis. In fact, cell competition plays a crucial role in tumor suppression, through elimination of early malignant cells, as part of Epithelial Defense Against Cancer. However, it is increasingly apparent that cell competition doubles as a tumor-promoting mechanism. The comparative nature of cell competition means that mutational background, proliferation rate and polarity all factor in to determine the outcome of these processes. In this review, we explore the intricate and context-dependent involvement of cell competition in homeostasis and regeneration, as well as during initiation and progression of primary and metastasized colorectal cancer. We provide a comprehensive overview of molecular and cellular mechanisms governing cell competition and its parallels with regeneration.

## Cell competition

**Fig. 1:**
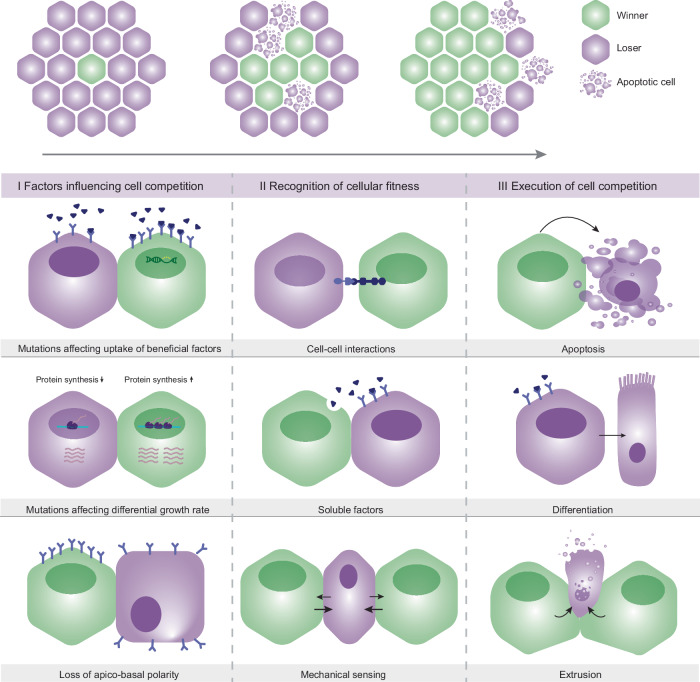
Schematic overview of the three stages of active cell competition. Cells acquire genetic alterations affecting their fitness levels. Relatively fitter cells become “winners” that actively force elimination of less fit cells (“losers”). **I** Induction of competition through mutations that can lead to differential uptake of beneficial factors from cells, mutations that can result in disparities of growth rates among cells and loss of apico-basal polarity that can compromise cell function and survival. **II** Cells sense differences in cellular fitness of their neighbors through several distinct mechanisms, such as direct cell-to-cell interactions, soluble factors released from “winner” cells and, the ability of cells to sense the mechanical pressure, under overcrowding conditions. **III** Pathways involved in the execution of cell competition. “Loser” cells can be either actively eliminated via apoptosis, forced to differentiation or extrusion from the epithelium.

During embryonic development, cells are subjected to a variety of pressures that may compromise their function and, subsequently, tissue integrity. Therefore, the effective elimination of abnormal cells is essential to ensure tissue fidelity and intact development of multicellular organisms. Upon cellular damage, cells activate a variety of innate stress response pathways that either promote cell survival or initiate programmed cell death [1]. Cell-based quality control mechanisms continuously guard the integrity of tissues and organs. These mechanisms are referred to as cell competition and consist of surveillance programs that can monitor relative cellular fitness levels among cells (Fig. 1). During cell competition, those cells perceived as less fit (“losers”) in a given microenvironment are eliminated, leading to the prevalence and expansion of the neighboring, fitter cells (“winners”) [1]. Cell competition was originally described in *Drosophila melanogaster* almost 50 years ago. Morata and Ripoll studied the effect of *Minute* mutations, which affect genes encoding ribosomal proteins, on the development of wing imaginal discs [2]. Although homozygous mutations of *Minute* are lethal, heterozygous *Minute* flies are viable and show a minor general developmental delay due to reduction in cellular proliferation rate. Interestingly, studies in genetic mosaics showed that *Minute*^−/+^ cells in a wild-type background cannot survive and are susceptible to apoptotic cell death while wild-type cells proliferate at the expense of the mutants [2, 3]. These findings suggest that relative differences in the proliferation rates of the two cell populations lead to cell competition. A similar pattern was observed in mouse chimeras, where cells bearing heterozygous mutations in the *L24* ribosomal protein gene exhibit reduced levels of protein synthesis and proliferation, are outcompeted by wild-type cells [4]. The concept that cell competition depends on the relative rather than the absolute levels of fitness between two neighboring cells is reinforced by the phenomenon known as “supercompetition”. According to this, wild-type cells can be eliminated if the surrounding cells pose a proliferative advantage over them [5]. This was first observed in the imaginal wing disc of *Drosophila*, where cells overexpressing the proto-oncogene *Myc* outcompete their wild-type neighbors that have a lower proliferation rate [6, 7]. Furthermore, studies in mosaic murine tissues, such as the epiblast and heart, showed that *Myc* overexpression results in elimination of adjacent wild-type cells [8–10]. These findings support that cell competition is a context-dependent process that optimizes tissue fitness (extensively reviewed in: [8, 11]). In this review, we describe the different modes of cell competition and highlight their roles in primary and metastatic colorectal cancer.

### Passive and active cell competition

Quality control of tissues plays a crucial role in maintaining organ function and two distinct modes of cell competition are involved in this: passive (either neutral or biased) and active competition. The first mode passively influences the potential of cells to self-renew without affecting surrounding cells and typically plays a role in stem cells, the pool of undifferentiated cells that is responsible for replenishing cells and ensuring that damage can be repaired [12]. Tissue turnover often involves cells with a similar genetic profile and comparable fitness level. Therefore, tissue replenishment is driven by stochastic loss and replacement of cells, which is known as neutral competition [13, 14]. The second passive type, biased competition, drives cell selection by favoring “winner” cells without directly interfering with surrounding cells. Even though genetically identical cells have an intrinsic comparable level of fitness, cell-extrinsic factors, such as location within a stem cell niche and availability of growth factors, can still determine their maintenance within a tissue [15, 16]. Alternatively, biased competition can be caused by genetic variations that result in minor cellular fitness differences between neighboring cells. Small differences in fitness result in a slight advantage for the progeny of that specific clone, which over time can cause one clonal population to overtake a niche [17–19]. In contrast, through active competition, the behavior of cells is directly influenced by neighbors with a differential fitness. This active cell competition can be considered as a stepwise process that requires cellular alterations that affect cellular fitness, the recognition of differences in relative fitness and cell selection. To decipher the factors that trigger cell competition, we should first unravel the key genetic alterations that can induce a gradient of fitness among cells. Such genetic alterations can globally be classified into three groups, which are addressed in more detail below (Fig. 1).

#### Disparities in proliferation rates

Several studies have illustrated that two competing cell populations are often characterized by a differential proliferation rate where fitter cells grow faster than loser cells. This is highlighted in the wing disc of *Drosophila*, where slow-growing *Minute* mutants are outcompeted by fast-growing wild-type counterparts [2]. However, growth differences among adjacent cells are not determinant for promoting elimination of the hypo-proliferative clones. For instance, overexpression of the pro-proliferative cell cycle regulators Cyclin D and cyclin-dependent kinase 4 (CDK4) does not induce elimination of surrounding wild-type cells. Instead, overexpression of such genes results in larger organs [6, 7]. Initially, protein synthesis was thought to be a main driver of cell competition, based on the decreased ribosomal translation that characterizes *Minute* mutants in *Drosophila*. However, a recent study shows that proteotoxic stress is the main cause of outcompetition of *Minute* mutant cells [20]. *Myc* overexpression has been correlated with both enhanced protein synthesis and aerobic glycolysis [6, 7] and in the mosaic wing disc of *Drosophila*, confrontation of *Myc*-overexpressing cells with wild-type cells enhances the metabolic switch of the former to glycolysis [6, 21]. Hence, *Myc*-overexpressing mutants acquire a “supercompetitor” status by reprogramming their metabolism due to their interaction with wild-type cells. Interestingly, in turn, both *Minute* mutants and wild-type cells in *Myc*-overexpressing mosaic wing discs experience low Dpp/BMP signalling levels [6], indicating that mutations compromising the relative cellular fitness can lead to reduced uptake of beneficial factors (Fig. 1). Together this suggests that differences in anabolic pathways and energy metabolism can induce competition among cells. Similarly, it could be argued that the metabolic reprogramming characterizing cancer cells, helps them to adopt a “supercompetitor” behavior that favors their progression at the expense of the surrounding healthy tissue.

#### Disparities in activation of signaling pathways

Several studies support that differences in signaling pathways that regulate cell proliferation and growth, such as WNT, can trigger competition among those cells [22]. In the wing disc of *Drosophila*, cells with low levels of WNT/Wingless are susceptible to elimination by wild-type counterparts. A second pathway that can trigger cell competition among cells with disparities in the signaling levels is the highly conserved JAK-STAT pathway. The effect of this pathway in cell competition was described in mosaic eyes and wing discs of *Drosophila*, consisting of *Stat92E*^−/−^ and wild-type clones. Interestingly, *Stat92E*^−/−^ clones are eliminated through apoptosis induced by wild-type cells that undergo compensatory proliferation [23]. Conversely, hyperactivation of JAK-STAT pathway allows cells to eliminate wild-type neighbors [24]. The Hippo pathway, implicated in cell proliferation and maintenance of tissue homeostasis, is an additional example that stimulates competitive interactions among cells. Reduced TEAD activity in in vitro murine fibroblasts results in their elimination by surrounding wild-type cells. Furthermore, in *Drosophila* and mouse, overexpression of YAP/Yorkie or Tead4 with the subsequent Hippo inactivation can turn the cells into supercompetitors that enforce programmed cell death of wild-type neighbors [25, 26].

#### Loss of apico-basal cell polarity and architecture in epithelial tissues

Cell shape is fundamental in regulating the morphology, migration, and functionality of many cell types and therefore has a major impact on defining and maintain tissue architecture. There is a high correlation between cell shape and polarity, which is the asymmetric distribution of cellular components. Defects in cell polarity can compromise tissue homeostasis by affecting cellular response to signals, intercellular communication and intrinsic processes, such as survival, proliferation, and migration [27]. In epithelial tissues, every cell is polarised and must form an apical surface, facing toward the outside of the tissue and a basal surface that is attached to the basal membrane. In *Drosophila*, homozygous mutations in any apico-basal polarity-related gene results in excessive tissue overgrowth and loss of tissue organization. Notably, these mutants within a mosaic background are actively eliminated by the wild-type neighbors, which implies that disruption of cell polarity can render cells unfit and, therefore, induce cell competition [28]. Loss of apico-basal polarity in *Drosophila* imaginal disk cells due to mutations in, e.g., *discs-large* or *scribble* results in mislocalization of TNF receptor Grindelwald. As a consequence, mutant cells become responsive to the circulating TNF ligand Eiger, while wild-type cells with correctly localized Grindelwald remain insensitive. This results in JNK activation and subsequent apoptosis-dependent elimination of polarity mutant cells from epithelia [29, 30].

### Fitness-sensing mechanisms

Upon induction of cell competition, the second and most important step that allows competition is the ability of cells to sense differences in fitness (Fig. 1). The transmembrane protein Flower constitutes a potential mechanism, by which cells could communicate their fitness status [31]. In fruit flies, *Flower (FWE)* gene encodes three isoforms, the “ubiquitous” or FWE^Ubi^ that is expressed throughout the wing disc and another two FWE^Lose-A^ and FWE^Lose-B^ isoforms that are upregulated in cells with a “loser” phenotype during cell competition [31]. Interestingly, this pattern was observed in mosaic tissues of *Myc*-overexpressing and *Scrib* mutants in *Drosophila*, where *FWE*^*Lose*^ expression was critical for “loser” cell elimination [32, 33]. Mammals have four FWE isoforms; FWE2 and FWE4 that act as super competitors and eliminate cells expressing FWE1 and FWE3 [34]. Furthermore, several tumor types express “winner” isoforms of FWE, which provides a growth advantage over the surrounding stromal cells that express the “loser” isoform [34]. Therefore, the “Flower code” seems to play a crucial role in cell selection by informing neighboring cells about their relative fitness status and, consequently, promoting the elimination of a less fit population. However, the exact mechanisms by which cells express and recognize the “Flower” proteins remain elusive.

In tissues, a high cell density can generate increased mechanical pressure through cell-to-cell and cell-to-extracellular matrix interactions, which can be released by balancing cell proliferation and elimination [35]. During mechanical cell competition, cells with superior mechanical properties enforce the elimination and delamination of inferior ones [36]. For instance, during notum development in *Drosophila*, oncogenic *Ras* mutations can cause cell overcrowding, which is sensed by surrounding wild-type cells. As a consequence, these wild-type cells are eliminated via apoptosis through compaction-driven downregulation of the EGFR/ERK signaling [37]. These studies suggest that cells have developed several mechanisms by which they can eliminate less-fit cells based on their mechanical properties.

### Execution of cell competition

After cells have evaluated their relative fitness, they should act according to their acquired “winner” or “loser” status (Fig. 1). This entails the elimination of less-fit cells through apoptosis, extrusion or forced differentiation. In parallel, cells with a higher fitness capacity can undergo compensatory proliferation and effectively fill the void left by eliminated cells. Although the mechanisms driving compensatory proliferation of winner cells are less clear, some of the pathways related to the elimination of unfit cells have been identified, including the well-studied stress response pathways JNK and p53. C-Jun N-terminal kinase (JNK) signaling was the first pathway that was closely tied to cell competition. JNK signaling is increased in heterozygous *Minute* mutants in mosaic wing discs, as well as in wild-type cells that were surrounded by *Myc*-overexpressing cells in *Drosophila* [3, 6]. In both cases, inhibition of the JNK pathway in cells that were regarded “losers” reverted their elimination. In the fly midgut *Apc* mutations turns cells into “supercompetitors” that eliminate the surrounding wild-type cell population in a JNK dependent manner [38]. However, the role of JNK signaling in elimination of unfit cells upon cell competition is less clear in other models of competition [7, 26]. Therefore, it is suggested that although JNK signaling has an important role in active elimination of unfit cells, it might not be the global key regulator of inducing their programmed cell death. In cell competition induced upon loss of cell polarity, JNK signaling irrefutably plays a crucial role in promoting apoptosis in unfit cells in both MDCK cells and *Drosophila* models [39, 40]. p53 constitutes a well-known stress response pathway that can enforce the elimination of unfit cells upon competition. For example, *Scrib* knockdown (*Scrib*^KD^) in MDCK cells results in elevated p53 levels, which causes them to become hypersensitive to crowding. Moreover, *Scrib*^KD^ cells become compacted, which leads to p38 activation, further increasing p53 levels. As a consequence, activation of p53 is a key factor that causes elimination of *Scrib*^KD^ through mechanical competition [41]. In *Drosophila* mosaic tissues, upregulation of p53 in fitter cells is required for their capacity to eliminate wild-type neighbors [7, 42]. This also indicates that p53 is required in precancerous cells for early survival and that they only later become p53 independent. Collectively, these are examples of mechanisms that are proposed to initiate cell competition, sense the relative fitness levels among cells and execute the context-dependent elimination of unfit cell populations.

## Cell competition in primary colorectal cancer

### Intestinal homeostasis

**Fig. 2:**
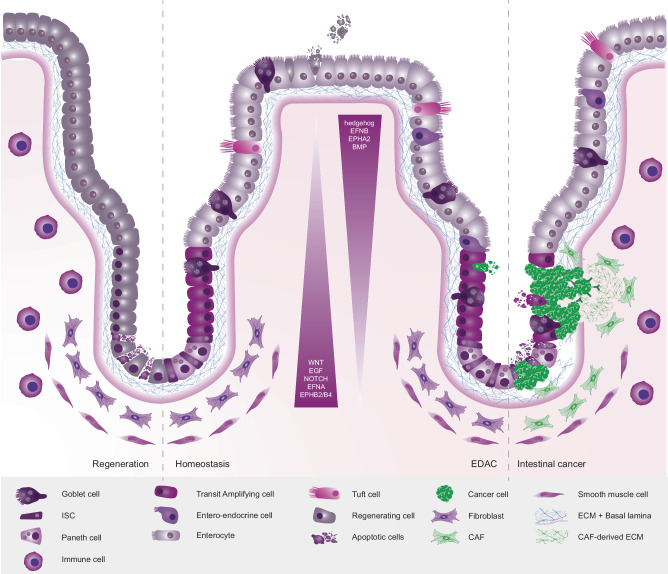
Schematic overview of the intestinal epithelium. **Regeneration:** Upon damage, all cell types in the intestinal villus and crypt can regenerate and repopulate. Regenerating cells activate a damage-response associated with specific gene expression as well as loss of differentiated morphology. Moreover, immune cells enter the tissue to combat inflammation. **Homeostasis:** Proliferation occurs in ISCs, and progeny differentiates as they move upwards along the villus. At the villus top, mature enterocytes are shed, maintaining a high cell turnover. In the center, gradients are shown as observed in homeostasis. **EDAC:** Early malignant cells are recognized by surrounding healthy epithelial cells and extruded locally from the tissue to prevent spread of cancer cells. **Intestinal Cancer:** When malignant cells are not recognized and extruded, they can progress. Upon gaining several oncogenic mutations, the cellular fitness of cancer cells increases and they gain the ability to eliminate surrounding wild-type epithelial cells.

Epithelial integrity is crucial for proper functioning of the intestinal tract. The epithelium acts as a selective barrier that allows absorption of nutrients and prevents pathogens from entering. To maintain tissue integrity, cells within the intestinal epithelium have a high turnover. Intestinal stem cells (ISCs) give rise to a variety of specialized cells that continuously replace the differentiated cells in the epithelium, which prevents accumulation of damaged and potentially harmful cells (Fig. 2). ISCs reside at the bottom of the crypt where they divide to give rise to Transit Amplifying (TA) cells. The progeny of TA cells undergo differentiation as they move along the crypt toward the villus. Most of the epithelium is occupied by enterocytes, which are responsible for selective absorption of ions, nutrients, and water from the intestinal lumen. They are characterized by their apical brush border of microvilli, maximizing their absorption surface. Enteroendocrine cells produce and secrete gastro-intestinal hormones such as secretins and gastrins to aid nutrient uptake, and e.g., ghrelin to signal to the enteric nervous system [43]. Tight junctions are crucial for maintenance of the epithelial barrier, as they are responsible for closely sealing adjacent epithelial cells and thus preventing leakage and pathogen entry [44]. This seal ensures that only selective transport can take place across the epithelial barrier. The epithelium itself is protected by a primary non-specific barrier that is formed by mucus produced by goblet cells under influence of STAT3 [45]. The intestinal mucus barrier is reinforced by a variety of enzymes and anti-microbial proteins such as α-defensins and lysozyme secreted by Paneth cells [45–48]. Paneth cells use this mechanism to defend ISCs and their location flanking ICSs at the bottom of crypts ensures that concentrations of anti-microbial proteins are highest near ISCs [49, 50]. An extra layer of protection is provided by tuft cells. They act as chemo-sensors and were shown to play a role in secretion of endogenous intestinal opioids and anti-inflammatory prostanoids such as cyclooxygenase [51, 52]. Besides their protective role, Paneth cells are required for development and maintenance of the stem cell niche [53]. The main additional components of this niche are CD34+ and Foxl-1 expressing mesenchymal cells [54, 55]. Lastly, highly specialized cells like enteric glial cells and macrophages have been reported to play a role in the intestinal stem cell niche as well, but their roles are currently poorly understood [56, 57].

#### Signaling during intestinal homeostasis

Communication between the different cellular components of the stem cell niche is crucial for maintenance of balanced proliferation in the crypt. WNT signaling is of vital importance for the maintenance of the ISC niche and through crosstalk with other signaling cascades plays a role in other processes such as crypt compartmentalization (Fig. 2). WNT ligands are secreted by mesenchymal and epithelial cells and WNT levels peak in the bottom of intestinal crypts, where it stimulates ISCs proliferation [58]. As ISC progeny travels toward the villus, the received WNT concentration rapidly decreases, causing a switch from proliferation to differentiation [59]. Besides spatial regulation, WNT signaling is modulated through secretion of WNT antagonists by Paneth cells. Interestingly, during aging secretion of the WNT antagonist NOTUM is increased, causing a reduced proliferative potential of old ISCs [60]. WNT signaling also directly regulates Paneth cells, but they respond differently to the same ligands due to differential expression of WNT receptors of the Frizzled family. Most ISCs express FZD5, but a subset of ISC progeny is primed to differentiate into Paneth cells through their expression of FZD6. This results in activation of the non-canonical WNT/PCP pathway, causing a cell-type specific response (Paneth- or ISC-specific) to WNT signaling [61, 62]. In addition, Paneth cells use NOTCH signaling to communicate with ISCs. This is supported by the observation that NOTCH signaling from ISC-progeny, such as secretory enteroendocrine cells to ISCs is required for the maintenance of multipotency in flies [63]. Moreover, levels of Notch-ligand Delta in fly ISCs determine daughter cell differentiation into enterocytes or enteroendocrine cells [64]. Genetic inhibition of NOTCH in mice showed its requirement for ISC proliferation and directs ISCs and TA cells toward a goblet cell fate [65]. Through inhibition of NOTCH signaling using γ-secretase inhibitors, which prevents cleavage of the NOTCH Intra-Cellular Domain (NICD) and subsequent downstream transcription regulation, the ISC marker *Olfm4* was identified as direct target of NOTCH activity [66]. Thus, NOTCH signaling is required for ISC proliferation and directs cell fate choice [67–69].

An additional stem cell niche-regulating pathway is EPH/Ephrin. The members of this protein family are transmembrane tyrosine kinase receptors (EPH) that regulate cell migration and tissue integrity through bidirectional signaling after binding ligands (Ephrins). EPH/Ephrin signaling is most active on the border between complementary gene expression domains, and in zones where multiple gradients overlap. In other words, EPH/Ephrin signaling is highest on the border between the EPHA-high and EPHB-high populations, which is matched by an inverse presence of EFNA and EFNB. EPH-Ephrin signaling results in repulsion of cells of different cell types or cellular fitness, and adhesion of matching cells or cell types [70, 71]. Moreover, EPHB signaling is required for niche maintenance and sorting of crypt and villus cells [72]. The genes encoding the EPHB2 and EPHB3 receptors are among the TCF-responsive genes, which means that intestinal EPH activity is indirectly regulated through WNT signaling [73]. Following the WNT gradient in the crypt, these two EPHB receptors are highly expressed in ISCs in the bottom of the crypt, while their ligand, EphrinB1, is exclusively expressed in the villus region. Thereby, the receptors and their ligands form opposing gradients that ensure crypt and villus cells remain segregated [74]. Paneth cells migrate toward the location with the highest WNT concentration using EPHB3 expression to move down the EphrinB1 gradient toward the bottom of the crypt, where they end up flanking ISCs [75].

Lastly, YAP/TAZ are crucial players in intestinal homeostasis. YAP/TAZ signaling is highly interconnected with WNT and EGF signaling. It directly regulates LGR5 expression in ISCs and controls self-renewal and progenitor expansion [76]. In fact, in YAP-deficient mice the ISC marker OLFM4 is downregulated, and crypt proliferation is dramatically reduced [77]. Moreover, deficiency of MST1/2 or LATS1/2, negative regulators of YAP/TAZ activity, results in expansion of ISCs and loss of secretory cells, corresponding to a shift in cell fate to the absorptive lineage [78]. ISCs respond to mechanical forces corresponding to cell density through YAP/TAZ [79].

### Intestinal regeneration

During homeostasis, the intestinal epithelium constantly regenerates to renew differentiated cell populations. In particular, enterocytes are short-lived (3–5 days) and need continuous replenishment. This high turnover is driven by the multipotent ISCs at the crypt bottom. However, during injury of the intestinal epithelium, homeostatic turnover is not sufficient and immediate repair and replacement of damaged cells is required to maintain tissue integrity.

#### Response to acute and chronic damage

Acute damage of intestinal epithelia can be mimicked by a variety of experimental strategies, such as ionizing radiation, and cell ablation using the Diphtheria Toxin Receptor (DTR) system. In the latter, the DTR is ectopically expressed in a cell type of interest, which renders them susceptible to Diphtheria Toxin (DT). For example, genetic engineering of the LGR5 locus was used to drive expression of the DTR in ISCs and treatment of animals with DT causes their ablation [80]. Exposure to ionizing radiation induces global damage in the intestinal epithelium, but it primarily affects proliferative cells. Paneth cells play a key role in this type of regeneration, by promoting proliferation in ISCs upon loss of enterocytes at the villus through NOTCH signaling. Besides the traditional LGR5+ ISC pool, additional reserve populations can contribute to intestinal regeneration. For example, a reserve pool of quiescent Tert+ stem cells are found around position +4 in the intestinal crypt. Upon exposure to ionizing radiation, these cells exit quiescence under the influence of WNT2b that is secreted by surrounding epithelial cells. Consequently, the Tert+ cells become mitotic and repopulate the damaged niche [81]. Interestingly, although the combination of radiation with DTR-driven ablation of LGR5+ cells reveals that radiation primarily affects LGR5− reserve stem cells, these cells hardly contribute to regeneration [82]. When damage to the ISCs is severe and these cells cannot repopulate the crypt, the tissue depends on alternative cell populations to sustain regeneration. Several different reserve stem cell pools have been described to fulfill this role. For example, quiescent p57+ tuft− and enteroendocrine precursors also dedifferentiate upon DTR-LGR5+ stem cell ablation [83]. Besides progenitors, Alpi+ enterocytes that reside inside the crypt region are reported to revert to highly proliferative stem cells that repopulate the niche [84]. Doxorubicin is a chemotherapeutic agent that inhibits topoisomerase 2 and specifically targets proliferative cells. Exposure to this drug causes a loss of ISCs, causing a population of Defa4-expressing Paneth cell progenitors to dedifferentiate and repopulate the ISC niche [53].

In contrast to reversible damage, inflammation can be permanent and result in a chronic damage response. A commonly used experimental method to mimic chronic diseases such as colitis and irritable bowel disease is prolonged treatment with dextran sulfate sodium (DSS). By combining induction of damage with lineage tracing experiments, it is possible to investigate the contribution of (reserve) progenitor pools, dedifferentiation and regeneration of the small intestine. For example, a progenitor pool destined to become Paneth cells and enteroendocrine cells can be activated to dedifferentiate upon exposure to epithelial damage or by receiving high NOTCH signaling, suggesting Paneth cells might be able to stimulate their progeny to alter their fate when required [85]. This population expresses stem cell markers like LGR5, but also enteroendocrine lineage markers such as MMP7 and Kit.

#### Signaling of the intestinal damage response

Both acute and chronic damage to the intestinal epithelium frequently results in reprogramming of epithelial cells to a dedifferentiated proliferative cell type. This reversion to a primitive state is often associated with an expression profile that largely overlaps with the transcriptome of spheroids generated from fetal stem cells [86, 87]. The similarity between colitis-induced damage response and embryonic tissue has also been observed on single cell transcriptomics level, suggesting a common response of reactivation of embryonic gene programs upon occurrence of intestinal damage [87]. Upregulation of this embryonic signature is highly conserved between human and mouse. The precise signature may be variable, which could be caused by differences in age, across cell types and or even type of damage induced. However, several key players seem to be involved in this damage response regardless of damage context. The signature is characterized by expression of genes of the *Ly6* family, *annexins*, and *clusterin* [88, 89]. It is also strongly associated with YAP activation [77]. In fact, YAP reprograms LGR5+ ISCs to an expanding LGR5- “revival” stem cell pool upon damage induction [77, 90]. This reprogramming causes inhibition of WNT-dependent proliferation and promotes cell survival of the epithelial cells. YAP/TAZ deficient intestines do not show this fetal-like damage response signature when exposed to damaging agents such as DSS, and subsequently do not regenerate properly [77, 91]. In stromal cells surrounding the stem cell niche, damage induces secretion of EGF ligands NRG1, AREG, hbEGF and EREG, which bind EGF receptors on ISCs, resulting in increased proliferation [77, 92]. Moreover, downregulation of the WNT pathway and upregulation of EGF can rescue ISC regeneration in YAP-deficient organoids, indicating that these two pathways downstream of YAP may act (partially) redundant in regeneration [77]. In most cases, the damage response is a temporary state, with one notable exception. Persistent immune activation due to infection with parasitic helminths results in activation of a gene signature in LGR5+ cells that is characterized by expression of *Ly6a*, but not *annexin* or *clusterin* [93]. Interestingly, when the in vivo damaged ISCs are grown in vitro, the spheroids remain cystic and show activation of this damage response gene signature long after isolation from the affected epithelium. This indicates that damage causes a long-lasting effect that extends beyond continued damage exposure. Taken together, intestinal regeneration is not just driven through proliferation of the stem cell compartment, but progenitors and even differentiated intestinal cells seem to be capable of repair, were it under the right circumstances. It has even been proposed that it is the sheer proximity to crypt bottom signals, that determines which cell type dedifferentiates and repopulates the crypt [17, 90]. Regeneration in the intestine is fueled from a highly diverse variety of cell populations, showcasing the intestine’s incredible plasticity (Fig. 2). However, it is important to note that many of these damage induction models do not necessarily represent intestinal damage as it occurs in vivo. Adequate regeneration of the intestine is vital for intestinal functioning. It is crucial to ensure that the regenerative proliferation program is abolished when the tissue has been replenished and when this inhibition does not happen at the correct moment, regeneration can eventually lead to tumor formation [94–97].

### Cell competition in colorectal cancer

Colorectal cancer (CRC) is the third most common cancer type and the second leading cause of cancer-related deaths in the world. In 2020, more than 1.9 million people were diagnosed with CRC and over 930,000 deaths were recorded worldwide [98]. CRC is characterized by an increased heterogeneity on a molecular and genetic level, which is highly correlated with the observed differences in clinical outcome and treatment response of the patients [99]. Approximately 70–80% of CRCs occur sporadically, whereas 20–30% of the cases are familial. Among the familial CRC cases, only 5% are attributed to highly penetrant inherited mutations that have been well characterized and are known as hereditary CRC syndromes [100, 101]. The cause of the remaining inherited CRCs, even though not yet fully understood, is likely linked to less penetrant alterations in multiple susceptibility genes regulated by environmental or other genetic factors [100]. In addition, chronic inflammation is associated with increased rates of CRC incidence. In fact, almost one fifth of patients with inflammatory bowel disease (IBD) have been reported to develop colitis-associated CRC [94, 102]. Colorectal cancer is often associated with loss of heterozygosity of WNT regulator APC, causing hyperplasia of the epithelium [103]. Tumor progression is driven by a subsequent accumulation of oncogenic mutations, such as activating mutations in KRAS and loss tumor suppressor genes such as Transforming Growth Factor β (TGFβ) and P53. During tumorigenesis, cancer cells are subjected to a remarkable array of intrinsic and extrinsic pressures that result in genetic diversification in the cell population. Over time, subclones with a distinct genetic composition undergo a constant competition (Fig. 2). Subclones, with features that increase their adaptation to the microenvironment, will survive and expand over unfit subclones leading to clonal selection [104, 105]. This dynamic process drives tumor heterogeneity akin to the theory of Darwinian evolution. However, limitations in tracing early neoplasms hamper the investigation of the exact with competitive mechanisms driving clonal evolution [106]. It is proposed that since cancer cells tend to reactivate developmental pathways, they can co-opt active competitive mechanisms that take place in developing tissues [107]. In line with this, an increasing number of studies supports that cancer development, progression and evolution depend on discrepancies in the relative fitness among cancer cells, as well as between cancer cells and the microenvironment [108].

#### Passive cell competition in primary colorectal cancer

Unlike during development, where apoptotic cell death is the main mechanism of cell competition, in adult tissues cell elimination is often mediated by inducing differentiation in stem cells through passive competition. In the intestine ISCs compete with their neighbors to prevail in a spatially confined niche. ISCs in the intestinal crypt continuously compete in a process called neutral drift, where stochastically arising neutral genetic variants spread through the stem cell compartment [15, 16]. Eventually, random ISC clones gradually take over the crypt, reducing clonal diversity until the crypt is monoclonal. Importantly, winning clones have no cellular fitness advantage over their neighbors, they win by sheer chance. Even though ISCs are equipotent and mainly undergo neutral competition, local concentrations of niche factors can affect competition among ISCs [17]. The architecture of the intestinal crypt creates a bias that is unrelated to their genetic background or cellular fitness. It generates a slight advantage for ISCs that are closer to niche cells, and thus in better reach of growth factors, or ISCs in contact with more Paneth cells, as NOTCH signaling from the Paneth cells is contact dependent. Reversely, in aging intestinal epithelia, niche cell contacts may provide a disadvantage, as aged Paneth cells show enhanced secretion of the WNT inhibitor NOTUM, which promotes the differentiation of the surrounding ISCs [60]. Therefore, ISCs surrounded by fewer Paneth cells, or ISCs that are located on average further away from Paneth cells, are more likely to self-renew. Moreover, the subsequent reduction of functional ISCs can affect regenerative capacity of the tissue, which may cause alterations in the dynamics of competition within the crypt. This may create a bias toward a specific ISC clonal populations or even promote clonal expansion of mutant cells. Not just the location relative to Paneth cells, but also the location along the crypt axis is a key determinant of ISC self-renewal. ISCs at a further distance of the crypt bottom are at higher risk of being physically pushed out by their neighbors and thus forced to differentiate. Moreover, as the peak of the pro-proliferative WNT signaling gradient can be found in the crypt bottom, some ISCs inevitably contribute more progeny as they will proliferate more. So, the otherwise neutral competition between ISCs somewhat favors ISCs in lower positions within the crypt, increasing their likelihood of fixing an entire crypt. Importantly, as biased competition is still a stochastic process, this means that less fit ISCs can also end up dominating a crypt. If a specific clone does acquire a cellular fitness advantage through mutation, tissue fixation can occur in a faster and more efficient manner [18, 19]. For example, gain of an oncogenic KRAS mutation by LGR5+ cells in the mouse intestine results in faster cell division, which sets the scene for oncogenic crypt fixation [18]. After crypt fixation, monoclonal oncogenic KRAS crypts expand through the intestine by crypt fission. In some cases, this results in a hijack of neutral drift. For example, once a crypt is fixated by an oncogene-expressing transformed ISC, the now-oncogenic crypt takes over neighboring crypts through two distinct paracrine signaling mechanisms [109]. First, through secretion of BMP ligands, the neighboring crypt’s ISCs are pushed toward differentiation by KRAS- or PI3K-expressing crypts. Second, PI3K-expressing crypts induce stromal cells to modify WNT levels at the bottom of the neighboring crypt. Together, these two mechanisms significantly speed up neutral drift.

#### Active cell competition in primary colorectal cancer

Epithelial defense against cancer (EDAC) is a process highlighting the impact of competition on suppressing tumorigenesis and is supported by the tendency of epithelial cells to suppress the outgrowth of mutant cells [110]. For instance, studies in flies have shown that cells carrying mutations in *Tp53* or *Src*, or overexpressing ERBB2 or YAP, could be extruded from different tissues [111–113]. In the murine intestinal epithelium, oncogenic HRAS expressing cells are rapidly eliminated through EDAC [114] (Fig. 2). Despite the protective capacities of EDAC, cancer does arise in the intestine with high incidence. Mechanisms determining the outcome of cell competition between wild-type intestinal tissue and intestinal cancer cells are not well-understood. Some studies suggest that the outcome is largely a numbers game. During cell competition as EDAC, initial mixing ratios are essential for the outcome [33, 114]. This is in stark contrast to mixed intestinal organoids, where mixing ratios, as well as degree of mosaicism, seem to determine the speed of competition, but not the outcome [115]. Turning of the proverbial tables can also be achieved when a cellular fitness discrepancy between wild-type tissue and transformed cells is decided in favor of cancer. This can occur either by lowering wild-type cell fitness or by increasing fitness of cancer cells. For example, a study in mice showed that diet affects the ability of the intestinal epithelium to extrude malignant cells [116]. In this study, mice were kept on a normal or a high-fat diet for 3 months, after which expression of oncogenic Ras^V12^ was induced in intestinal or pancreatic epithelial cells. In mice on a normal diet, Ras^V12^ transformed cells were apically extruded through tumor-suppressive cell competition. In contrast, in mice on high-fat diet the metabolism of epithelial cells changed, as did their cellular fitness, abolishing their ability to outcompete malignant cells. As a result, apical extrusion does not occur, enabling the malignant cell to persist and proliferate. Moreover, chronic inflammation also impairs apical extrusion of Ras^V12^ cells [116]. Taken together, this demonstrates that maintaining a high cellular fitness level in wild-type epithelial cells is essential to eliminate malignant cells and prevent further tumor progression.

When intestinal cells fail to extrude transformed cells, the subsequent cancer cell expansion is not the result of the cancer cells adopting a winner status, rather a failure of the wild-type cells to do so. In contrast, during supercompetition, competitive interactions and mechanisms are specifically hijacked by cancer cells to eliminate healthy cells from the tissue. Cancer expansion can be promoted by eliciting release of growth-promoting factors from wild-type cells by forcing them to differentiate. This can be achieved in two different modes. First, cancer cells can acquire mutations rendering them independent from niche growth factors. Examples of this are mutations in WNT signaling components such as APC, resulting in WNT-ligand insensitivity [117, 118]. These tumors no longer depend on pro-proliferative WNT signaling, as in these cells β-catenin is never degraded and WNT signaling is constitutively active. Second, loss of APC directly suppresses proliferation of surrounding wild-type ISCs through secretion of WNT antagonists such as NOTUM [119, 120]. Third, in RNF43 tumors, a subset of cells is forced to functionally specify into niche cells, secreting growth factors and mucus, which the tumor uses to boost its own growth [121]. Often, supercompetition results in increased cancer expansion, for example because of increased space availability through wild-type elimination. Active supercompetition by tumors was first shown in adult tissues in the *Drosophila* gut, where APC-deficient adenoma cells eliminate neighboring wild-type cells through induction of apoptosis [38]. Protection of wild-type cells by expression of apoptosis inhibitors, prevents cancer cell expansion. This highlights the dependency of cancer cells on a growth-permissive environment for colonization of tissues. Similarly, in mixed murine intestinal organoids wild-type cells are actively eliminated by APC^-/-^, P53^R172H^, KRAS^G12D^ mutated cancer cells in a JNK-dependent manner [115]. Remarkably, during competition wild-type ISCs are lost from mixed organoids. The remaining wild-type population reverts to a primitive state that is very similar to the fetal-like damage response, marked by expression of genes of the *annexins* and *Ly6* families. It is however unclear if that is a direct consequence of cell competition, or a consequence of loss of ISCs through cell competition. Together, this shows that the mechanisms and responses that are inherent to the intestinal tissue can be used by cancer cells to drive their progression.

## Cell competition in the liver and during liver metastasis

### Liver homeostasis

**Fig. 3:**
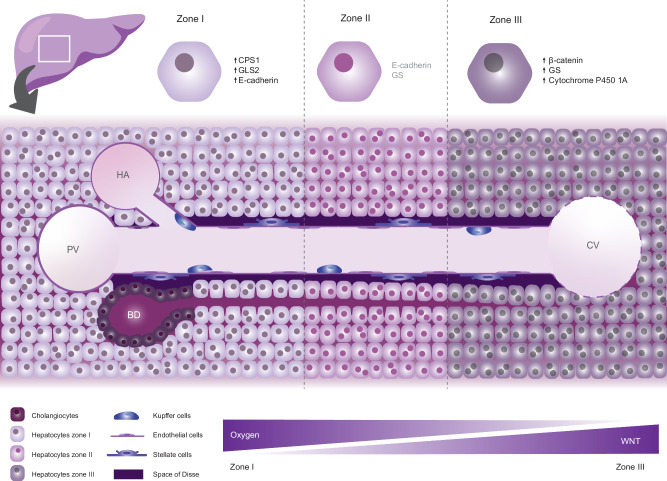
Schematic overview of the cellular composition and zonation of the liver in homeostasis. The liver is divided in three zones; Hepatic arteries, the portal veins and bile ducts are found closest to Zone I. Hepatocytes in zone I are exposed to the highest oxygen levels and are in the lowest WNT state. The transition area, Zone II, is where hepatocytes are characterized by absence of Zone I markers. Zone III hepatocytes surround the central vein and receive lower oxygen and nutrient input. Blood vessels are fenestrated, enabling exchange of nutrients, waste and oxygen. Liver resident macrophages (Kupffer cells) reside in the blood stream and stellate cells placed between the endothelium and hepatocytes in the space of Disse perform more general functions during development, regeneration and angiogenesis. Bile produced by hepatocytes is collected in the canaliculi between hepatocytes, and is accumulated in the bile ducts, which are surrounded by cholangiocytes.

Approximately one-third of colorectal cancer patients develop metastases within the first 3 years after diagnosis. The liver is a main site of colorectal cancer metastasis and is responsible for most of the colorectal cancer-dependent lethality. Therefore, we here focus on competitive interactions that occur in this organ. The liver is crucial for a plethora of processes that govern organismal physiology, such as metabolism, detoxification, regulation of blood clotting factors and bile synthesis [122]. This epithelial organ is predominantly quiescent, in comparison to other epithelial tissues such as intestine and skin [123]. However, upon damage such as acute injury or chronic inflammation, it shows a remarkable regenerative capacity [122, 124, 125]. The liver tissue comprises various cell types distributed around the hepatic architecture, including those surrounding endothelial ducts connecting the portal and central veins and those associated with bile ducts. Among the former are Kupffer cells, liver-resident macrophages, which form the liver's first line of defense against infections [126] and sinusoidal endothelial and stellate cells, which play crucial roles in liver regeneration and are responsible for synthesis of all extracellular matrix (ECM) components, as well as production of key signaling proteins [127–130]. The bile-duct associated cells are primarily hepatocytes and cholangiocytes, which together form the epithelial surface of liver tissue [125] (Fig. 3).

Hepatocytes constitute 60% of the cells and 80% of the volume of the liver [125]. They display a characteristic distribution in the tissue, known as “zonation”, and are typically divided in three zones. The division depends on their position relative to big vessels, and consequently oxygen availability in the environment [131, 132]. Zone 1 hepatocytes are located close to the portal vein and hepatic arteries, which means they have easy access to highly oxygenated and nutrient-dense blood. Zone 1 hepatocytes express markers as urea cycle genes (*Cps1*), glutamine catabolism gene (*Gls2*), and E-cadherin and oversee important metabolic processes as gluconeogenesis, lipid metabolism and ureagenesis [133, 134]. Hepatocytes in zone 3 are found near the central vein and express markers as glutamine synthase (*GS*) and members of the cytochrome P450 family such as *Cyp1a2*. These cells use glycolysis and the TCA cycle for energy production and are responsible for Glutamine synthesis and Xenobiotic metabolism. Zone 2 contains those hepatocytes that are located in between zones 1 and 3 (Fig. 3). They display an intermediate expression profile and can be recognized by the lack of expression of markers of both zone 1 (E-cadherin) and zone 3 (GS). Additionally, zonation of hepatocytes is correlated to LGR5 expression and WNT signaling. Areas close to central vein (zone 3) express LGR5 together with activated β-catenin, while LGR5 is reduced around periportal veins (zone 1) overlapping with the expression of negative regulator *Apc*, demonstrating a gradient of WNT/β-catenin signaling between zones [135]). Especially WNT2 and WNT9b are crucial in this distinction [122, 131, 133, 134, 136, 137] (Fig. 3). *Alb* and *Cyp3a11* are markers of mature hepatocytes, therefore, their expression level is a good predictor of functionality of the cells [138]. In addition, transcription factors of the HNF and C/ep families are required for liver function. For example, HNF4α one of the most common hepatocyte markers, is essential for hepatocyte differentiation and control of lipid homeostasis [139].

Cholangiocytes encompass a smaller number of liver cells compared to hepatocytes and form 3–5% of the total liver cell population [140]. They are needed for bile formation, one of the main functions of the liver, which is required for digestion of lipids. There are different types of cholangiocytes that can be classified based on their location in the distinct biliary tracks (intrahepatic and extrahepatic) and unique marker expression patterns [141]. All cholangiocytes express high levels of the transcription factor SOX9 and cytokeratins KRT9 and KRT7 [142]. However, cholangiocytes from the extrahepatic biliary tree, including gall blader cholangiocytes and common bile duct cholangiocytes, express aquaporins, mucins, FGF19 and SOX17, while intrahepatic bile duct-derived cholangiocytes express JAG11, TACSTD2, and several YAP target genes, corresponding with their localized functions [143]. Interestingly, organoid cultures initially show expression patterns, functionality and expansion potential that is similar to their area in the biliary tree of origin. However, specialization to other cholangiocyte types can be induced by addition of environmental cues to cultures. For instance, addition of bile to the cultures enhances specialization toward gallbladder cholangiocytes [143, 144], illustrating the plasticity of this cell population. In addition, the morphology and size of murine cholangiocytes changes depending on their location in the biliary tree, size of the duct and state of the tissue [140, 145, 146]. For example, acute damage induces the appearance of small, less differentiated LGR5+ cholangiocytes that can differentiate into larger cholangiocytes [147], and can even act as hepatic progenitors [148].

### Liver regeneration

#### The role of hepatocytes in regeneration

During liver damage, in particular acute injury caused by for instance viral infection, drugs and acute ischemia, the tissue switches from a quiescent to a regenerative state [149]. At this stage, regeneration is mostly driven by phenotypical fidelity, which means that each cell type is responsible for repopulation of their own cell type within the tissue [127, 150]. Therefore, since hepatocytes are the most abundant cell type in the liver, they play a critical role in acute damage regeneration. Acute liver injury is often experimentally modeled by partial hepatectomy, where a large part of the liver is surgically removed. Although the organ cannot reconstitute lost lobes, it will compensate by increasing the size of remaining lobes [123]. Lineage tracing experiments after partial hepatectomy showed increased proliferation of hepatocytes [122, 137, 151]. In addition, increase of hepatocyte size known as hypertrophy, can restore the mass of functional liver tissue during regeneration after acute injury [152]. Alternatively, acute liver injury can be induced by exposure to carbon tetrachloride (CCl4) or N-acetyl-para-amino phenol, which both cause liver toxicity. Interestingly, CCL4 administration causes loss of regenerative-prone pericentral vein LGR5-expressing hepatocytes. This damage is resolved by repopulation driven by LGR5- hepatocytes that switch to a LGR5+ population [153]. Lastly, HNF4α, crucial not only for hepatocyte function but also for liver development is required to terminate liver regeneration [139]. The expression of this transcription factor is downregulated in the first hours after damage and re-expressed at the end of regeneration to complete the process [122, 154]. After regeneration, zonation is restored by zone 2 hepatocytes that give rise to zone 1 hepatocytes when there is periportal injury and to zone 3 hepatocytes during pericentral injury [132]. Collectively, hepatocytes assume a key role in liver regeneration following acute liver injury. They undergo reactivation from their quiescent state, acquire proliferative capacity and increase in size. This underscores the significance of environmental signals in modulating the fitness of a particular cell type within a population of damaged cells.

#### The role of cholangiocytes in regeneration

Cholangiocytes play a vital role in regeneration when the hepatocyte-driven injury-response is impaired, for instance, when there is continuous tissue damage caused by chronic liver disease or severe liver injury. During such conditions, hepatocyte-fueled regeneration is compromised, and proliferation and subsequent differentiation of cholangiocytes becomes increasingly important. For example, the loss of integrin β1 in damaged hepatocytes initiates a ductal reaction with cholangiocyte origin, resulting in non-hepatocyte derived hepatocytes [141, 155]. In addition, lineage tracing studies have shown that experimental induction of chronic damage by repetitive administration of CCl4 and thioacetamide, or diets supplemented with 3,5-diethoxycarbonyl-1,4-dihydrocollidine (DDC) [122, 156] cause reactivation of cholangiocytes and subsequent differentiation into hepatocytes [155, 157–159]. Importantly, in vitro differentiated cholangiocyte organoids can repopulate the mouse liver with hepatocytes upon transplantation [148, 160]. It is still unclear whether cholangiocyte differentiation is sufficient to reconstitute the totality of lost tissue, but it is considered an important contributor, especially during chronic damage [123]. Interestingly, cholangiocytes can also undergo senescence upon chronical liver injury [161] and cholangitis [162]. This suggests that the regenerative response of these cells depends on the type and severity of the damage.

#### Bidirectionality of transdifferentiation

Transdifferentiation is not limited to the generation of hepatocytes from a cholangiocyte-origin, but instead is bidirectional: cholangiocytes can also arise from hepatocytes. Several studies show dedifferentiation of hepatocytes during liver injury such as non-alcoholic steatohepatitis [163], DDC-treatment [164] and alcoholic hepatitis [165]. Transdifferentiation coincides with an increased expression of cholangiocyte markers in the reprogrammed hepatocytes [164, 165]. This hepatocyte to cholangiocyte reversion is not limited to chronic damage and has also been observed during in vivo regeneration [166] and in vitro assays [167, 168]. Since this transdifferentiation can be bidirectional, cells in damaged mammalian livers can co-express both hepatocyte (e.g., HNF4α) and cholangiocyte (e.g., KRT19 and SOX9) markers simultaneously. Even if certain hepatocyte features remain, cells switch from a hepatocyte to cholangiocyte gene expression profile during transdifferentiation [129, 159, 164, 169, 170]. This illustrates the remarkable plasticity of the liver and the interconnected role of hepatocytes and cholangiocytes during liver damage. Furthermore, these observations emphasize that hepatocyte- and cholangiocyte-driven regeneration should not be considered as independent processes.

#### Molecular pathways involved in regeneration

To understand liver regeneration, insight into the involved molecular pathways and signaling is crucial [128]. WNT/β-catenin signaling is required for liver zonation [136, 144] and it also plays an important role in regeneration during early injury response. Activation of this pathway increases hepatocyte proliferation through ZNRF3 and RNF43 [171, 172] and transdifferentiation of hepatocytes into cholangiocytes [122, 128]. For instance, WNT-expressing *Lgr5+*, *Gs+* and *Axin2+* hepatocytes found in zone 3 [144] can self-maintain in homeostasis and are responsible for regeneration during hepatectomy. However, they are also more susceptible to transformation and subsequent induction of hepatocarcinoma due to misregulation of cell division [153]. Furthermore, activation of WNT signaling is not limited to hepatocytes [148], as damaged LGR5+ cells, which can regenerate hepatocytes and bile ducts, can be found in areas near bile ducts upon damage. These LGR5+ cells, derived from murine or human damage-induced livers, can form liver organoids in vitro when cultured in RSPO containing medium through activation of WNT signaling [148, 160].

Another key controller of hepatocyte and cholangiocyte function is YAP/TAZ signaling [173]. This pathway is a master regulator of cell proliferation and can integrate extra-cellular stimuli such as cell density and stiffness of the environment. YAP/TAZ signaling was originally identified by its role in regulation of organ size in *Drosophila* [174] and it fulfills a similar role in the mammalian liver [173]. During homeostasis, hepatocytes remain quiescent, keeping YAP expression low. Indeed, deletion of this pathway in adult hepatocyte does not cause a phenotype [175, 176]. However, increased expression of YAP in hepatocytes leads to loss of hepatocyte identity and conversion to cholangiocytes [122, 173]. The plasticity of this process is illustrated by the finding that reduction of this elevated YAP promotes redifferentiation into hepatocytes [177]. In vitro, YAP activation can be triggered by mechanical tension and formation of stress fibers in hepatocytes, causing dedifferentiation and subsequent loss of hepatocyte function [173, 178]. Moreover, YAP signaling is needed for liver regeneration both after acute and chronic liver injury. Downstream signaling of YAP such as NOTCH, induces hepatocyte to cholangiocyte transdifferentiation upon damage [173, 177]. After acute injury, YAP activity is increased at the first day of regeneration and decreases afterwards [179]. Importantly, liver-specific deletion of both YAP and TAZ, through albumin-Cre-mediated recombination, causes major delays in liver regeneration post-hepatectomy [180]. Similarly, deletion of YAP/TAZ impairs cholangiocyte dependent regeneration during acute [181] and chronic injury [172, 173]. Together, this shows that YAP/TAZ activation is required for liver regeneration after acute injury, especially during cholangiocyte-dependent regeneration.

### Cell competition in liver transplantation

Given the liver’s innate regenerative capacity, one of the investigated strategies for mitigating liver damage involves the transplantation of healthy hepatocytes into compromised tissue. Cell competition plays a key role, as the objective is to enable outcompetition of damaged cells by more robust counterparts, ultimately leading to functional tissue repopulation. Already in 1994 a study showed that transplanted adult liver cells into an adult damaged mouse liver replaced 80% of the damaged organ. To follow repopulation in the liver, mature hepatocytes were injected in mice overexpressing the hepatotoxic transgene urokinase plasminogen activator (uPA). After 2–3 weeks the damaged livers showed a colonization of healthy infused cells, while they were absent in control mice lacking the uPa transgene [182]. After that, a plethora of publications supported the potential of liver transplantations in liver regeneration after damage [183–186]. Thus, fitter transplanted cells can outcompete damaged cells and repopulate the liver. A similar observation is made when tissue fitness is reduced by radiotherapy post-surgical resection. Transplantation of healthy hepatocytes in such livers promotes the regeneration of damaged liver tissue and restores its functionality [183]. This phenomenon was substantiated by the combination of radiation with ischemia-reperfusion, where intra-splenic transplanted healthy hepatocytes facilitated the regeneration of the compromised tissue [187].

**Fig. 4:**
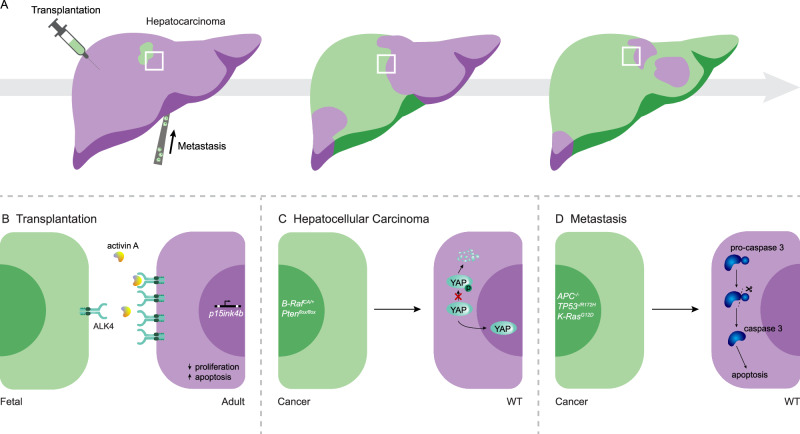
Schematic overview of competitive interactions in the liver. **A** Examples of three types of cell competition that play a role in the liver, resulting in elimination of weaker cells (purple) and subsequent colonization of the liver by fitter cells (green). **B** During transplantation adult hepatocytes are outcompeted by newly introduced hepatocytes that colonize the liver. Loss of regenerative capacity is associated with a rise in Activin A-mediated growth inhibition. **C** Through accumulation of mutations, hepatocellular carcinoma cells gain a fitness advantage over non-mutated neighbors, resulting in tumor expansion. In response, wild-type peritumoral hepatocytes activate YAP signaling in attempt to control tumor progression [194]. **D** Intestinal cancer cells interact with wild-type hepatocytes, resulting in induction of apoptosis [216].

The competitive advantage exhibited by healthy liver cells over their defective counterparts can also be harnessed for gene therapy. For example, livers from animals suffering from Hereditary Tyrosinemia Type I are repopulated by wild-type liver cells upon transplantation [184, 186]. This suggests that transplantation of fitter cells in a less fit recipient liver will lead to competition that promotes repopulation to restore its functionality (Fig. 4B). Furthermore, when fetal liver cells from healthy rats were transplanted into DPPIV-F344 mutant livers via portal vein injection after partial hepatectomy, they exhibited higher proliferation rates compared to the host liver cells. Interestingly, the recipient liver cells display increased apoptosis in areas surrounding the repopulated tissue, hinting that the elimination of host cells was triggered by transplanted fetal cells [188]. This elimination and repopulation is driven by a differential sensitivity to Activin A signaling, which induces apoptosis in older hepatocytes, while fetal cells are resistant and remain proliferative and resemble active cell competition [189, 190]. These findings display the potential of utilizing cellular fitness to improve human liver transplantation, however, a major complication is potential tumorigenesis due to misregulation of cell competition during repopulation [191]. For instance, retrorsine, a proliferation blocking alkaloid, causes reduced fitness of liver tissue and a massive proliferation of transplanted normal hepatocytes that can result in development of hepatocellular carcinoma [185, 192]. In contrast, no aberrant growth is observed after hepatocyte injection into non-retrorsine treated recipients. Similar tumor-promoting effects are observed in other models of liver repopulation reviewed in [193]. Together, this shows that the host microenvironment during transplantation plays a deterministic role and, cell competition can control the growth of transplanted liver cells. This implies that an imbalance in cellular fitness can lead to a desired outcompetition of the less fit host tissue, but also facilitate overgrowth and subsequent tumor formation by fitter transplanted cells.

### Cell competition in primary liver cancer

Overgrowth of fittest cells in the liver is not an exclusive consequence of transplantation. Competing interactions within the tissue also take place in homeostatic conditions without liver damage. However, when liver cells acquire mutations which provides a phenotypical advantage over neighboring tissue, such as higher proliferation, these mutated cells can overtake the tissue. If this repopulation takes place in an uncontrolled manner, it will result in liver tumor growth (Fig. 4C). Interestingly, there is a large overlap in mechanisms that are important for regulation of regeneration and those that are needed for growth restriction. A key example of this in hepatocellular carcinoma, is YAP/TAZ signaling, which plays an important role in regulation of hepatocyte fate during regeneration [177]. Importantly, peritumoral hepatocytes activate YAP and TAZ, thereby inhibiting tumor growth. Conversely, when these mechanisms are disrupted or deleted, tumor progression is accelerated [194]. Therefore, YAP and TAZ show an important role in competition between tumor and healthy liver cells to control tumor growth.

### Cell competition in liver metastasis

The liver is one of the most common sites of colorectal cancer metastasis. A major underlying cause is the location of the organ within the vascular system, as the blood circulation from the intestine directly drains into the portal vein which serves as the principal entry point to the liver [195]. Moreover, the hepatic vasculature is characterized by fenestrations and a lack of subendothelial basement membrane, which allows cancer cells to escape from the blood circulation and colonize the liver niche [195–197]. The metastatic cascade globally occurs in four steps. First, during the “tumor infiltrated microvascular phase” cancer cells reach the microvasculature and get trapped in sinusoidal vessels. If these cells survive, they advance to the subsequent stage “extravascular or pre-angiogenic phase”, which refers to extravasation of cancer cells. During this second step, cancer cells access the perisinusoidal space (or space of Disse) by endothelial transmigration. Here, they employ stellate cells for ECM deposition and release of angiogenic and growth factors, thereby setting the groundwork for tumor progression. Next, during the third step or “angiogenic phase”, cancer cells increase expression of adhesion molecules and promote vascularization for sufficient oxygen and nutrients supply. Lastly, in the fourth step or “growth phase”, tumor angiogenesis will facilitate expansion from micro-toward macro-metastases [198].

In the liver, metastases exhibit diverse growth patterns, each distinguished by distinct histo-pathological traits (see [199] for a recent extensive overview). Notably, these distinct growth patterns engage various cellular interactions, and therefore some are more prone to be regulated by cell competition. Both the desmoplastic and pushing growth patterns, are characterized by a sharp well-defined edge of the metastasis, compression of surrounding liver cells and absence of liver mimicry. The difference between the growth patterns is the stromal barrier between cancer cells and liver tissue, which is only observed in desmoplastic metastases. Consequently, both growth patterns show tumor infiltration of immune cells, however other stromal cells are lacking in metastases with the pushing growth pattern. The replacement growth pattern, on the other hand, is very different from the other patterns as it is characterized by a high degree of cellular interactions between liver and cancer tissue. Cancer cells grow within plates of hepatocytes, display a high level of liver mimicry and preservation of stroma. In this pattern, cancer cells are in contact with and progressively replace the surrounding hepatocytes. By these means, replacement-type metastases can co-opt the existing sinusoidal vessels of the liver for blood supply, resulting in minimal hypoxia compared to desmoplastic liver metastases. The borders of metastases with a replacement pattern lack a sharp contour and are generally irregular. Importantly, clear contact between hepatocytes and tumor cells is observed. Some tumor infiltration can be found, but in general, the replacement pattern is characterized as immune desert both at tumor-liver interface and central part of the tumor. The exact biological mechanisms driving the establishment of a certain metastatic growth pattern remains elusive. Several studies suggest that replacement is the default growth pattern upon initiation of liver metastasis [199]. This is supported by the detection of structures that are typical for the replacement pattern in desmoplastic liver metastases. This indicates that replacement precedes the emergence of the desmoplastic growth pattern, which relies on spontaneous or induced transition [199]. The behavioral similarities with the hepatic tissue, renders replacement-type as the most aggressive growth pattern and highlights the tendency of cancer cells outcompeting the less aggressive and weaker surrounding hepatocytes [200]. This reinforces the need to comprehend the intricate interactions between cancer and liver cells and give insight into pathways that could be targeted for therapeutic purposes.

#### Passive competition in liver metastasis

Hepatocytes are a source of soluble factors that promote metastatic growth. They release growth factors like IGF-I and HGF-like protein/macrophage-stimulating protein which enhance tumor growth, motility, and invasiveness. For instance, liver-circulating HGF binds c-MET in cancer cells promoting growth of liver metastases by regulation of a wide range of downstream pro-metastatic pathways such as JAK2-STAT3 signaling, Ras-MAPPK-ERK and PI3K-AKT [201]. This pro-metastatic support can be further enhanced by hypoxia. Under hypoxic conditions, factors like HIFs, VEGFs, and G-CSF are induced, which support the colonization of cancer cells in pre-metastatic tissues [202, 203]. Besides soluble factors, also modification of the amount and composition of ECM is crucial for metastatic growth, and it is often enhanced by resident immune cells. In the liver, Kupffer cells drive fibronectin synthesis and deposition by releasing TGFβ, which activates fibroblast-like hepatic stellate cells. Upon activation, stellate cells can enrich the ECM in collagens I and IV, thereby promoting liver fibrosis which subsequently enhances tumor immune evasion and angiogenesis [204]. Liver fibrosis provides a supportive environment for metastatic colonization. For instance, patients with colorectal cancer and fibrotic livers exhibit higher metastatic incidence and worse survival rates [205, 206]. Interestingly, these processes can be modulated by tumor cells both prior and during metastasis. It is shown that a pre-metastatic niche, that is established by secreted factors stemming from the primary tumor, contributes to the transition of the liver microenvironment from healthy to tumor-receptive [207]. Tumor-derived secreted factors and extracellular vesicles are potential mediators for fostering this inflammatory pre-metastatic niche by either recruiting tumor-promoting bone marrow derived cells to metastatic sites or mediating the crosstalk between tumor cells and the surrounding host cells [208, 209]. In addition, upon arrival in the liver tumor cells face mechanical stress of sinusoids and the cytotoxic activity of immune cells, resulting in the apoptotic death of some metastatic cells. This creates an inflammatory state that enhances the expression of adhesion receptors in liver-resident cells that facilitate cancer cell mobility and migration in liver tissue [210, 211]. Together, these findings highlight the pro-metastatic role of liver tissue and suggest potential passive mechanisms of cell competition that can be used by cancer cells to promote metastatic growth.

#### Active competition in liver metastasis

Besides passive mechanisms, active competitive interactions take place between metastases and surrounding liver tissues. Upon arrival in liver, circulating cells can adapt to cues from the local microenvironment. For instance, PRL3 is specifically activated in CRC liver metastasis where it induces several pro-metastatic cascades. PRL3 promotes invasion through activation of the AKT pathway [212], enhances angiogenesis via the NK-κβ pathway, and leads to proliferation and invasiveness by activation of STAT3 signaling. This suggests that, upon their arrival in the liver, cancer cells receive cues from the surrounding tissue that enhance invasion and causes increased fitness through PRL3 activation [201]. Similarly, NOTCH activation is recurrently found in CRC liver metastasis, suggesting that liver microenvironment provides cues that activate this pathway. Interestingly, this has a dual effect as high expression of NOTCH 1 or 3 correlates with a poor prognosis while overexpression of NOTCH 2 or 4 has a protective function by blocking proliferation, invasion and migration [213]. Besides intrinsic changes to cancer cells, competitive interactions can also be used to impact surrounding liver tissue. A key example in colorectal cancer liver metastasis is vessel co-option, the utilization of pre-existing vessels by proliferating cancer cells [214]. This particularly prevalent metastases of the replacement growth pattern and is crucial for nutrient-supply and oxygenation. Furthermore, the CD95 (Fas-Ag/ Fas-L) pathway drives colorectal cancer tumor progression and invasiveness. Fas-L expressing cancer cells induce apoptosis in Fas Ag-bearing hepatocytes, creating a favorable niche for growth metastases in liver tissue [215]. Furthermore, in microtissues, an in vitro model for liver metastasis, intestinal cancer cells induce apoptosis of wild-type hepatocytes [216]. This resembles processes of active cell competition where tumor cells eliminate surrounding healthy tissue [38, 115] (Fig. 4D). Interestingly, signaling pathways that drive competitive interactions during transplantation, might also play a role in liver metastasis. For instance, expression of the Activin A receptor ACVR2A is reduced in primary tumors and metastases, and this correlates with lympho-vascular invasion [217–219]. This leads us to hypothesize that low expression of ACVR2A by metastatic cancer cells renders them less susceptible to Activin A signaling upon arrival in the liver. In that way, they could potentially outcompete the wild-type liver cells in a mechanism that parallels the increased competitive advantage of young hepatocytes. Lastly, differences in the expression levels of YAP can induce competition between metastatic lesions of melanoma and colorectal cancer in liver and the surrounding healthy tissue [194]. As this is a tumor-preventive cell competition mechanism in primary hepatocellular carcinoma this suggests that similar mechanisms are important in liver metastasis [194]. An additional study illustrated that upon arrival of differentiated cancer cells in liver, high YAP levels mediate their conversion to cancer stem cells, a crucial cell population for supporting survival and outgrowth of liver metastases. However, the subsequent, gradual decrease in YAP levels of cancer cells is key for the establishment of cell heterogeneity, a determinant for tumor progression [219]. Taken together, these studies suggest that at initial stages, dynamic YAP expression is important for the establishment of metastatic lesions in the liver with their future survival and outgrowth relying on relative YAP expression levels between cancer and healthy cells.

Thus, a broad spectrum of pathways is activated in cancer cells upon arrival in the liver and, vice versa, this inflicts crucial changes within the liver microenvironment, together facilitating the formation of metastases. The complete extend of such interactions and the interplay between different types of competition during metastasis remain elusive. For instance, it is unclear whether liver colonization starts with passive competition which can eventually lead to an increased competitive potential that allows active elimination of wildtype cells in evolving niche. Furthermore, the presence of activated damage/repair programs in the liver can enhance the competitive advantage of colorectal cancer liver metastasis, however, whether the formation of liver metastasis is exclusively dependent on this is unclear. Therefore, unraveling these pathways and the cues, factors, or ligands furnished by the liver microenvironment—whether originating from cellular or non-cellular components—holds promise for discovering new therapeutic targets to impede CRC liver metastases.

## Discussion

The multifaceted role of cell competition in cancer underscores the need to further understand what determines winner or loser status. Generally, whether tumor or wild-type is outcompeted is context-dependent, illuminating the complexity of cellular interactions within distinct tissues. While cell competition safeguards against tumor initiation in the homeostatic intestine, its paradoxical role stimulating the growth of colorectal cancer highlights the intricate interplay between genetic and microenvironmental factors. For instance, loser populations share many characteristics with regenerating epithelia, which is an important aspect that is normally observed upon acute injury to protect the epithelium. The relationship between regeneration and supercompetition, is essential to comprehend the communication between cancer and the wild-type tissue. This allows a better understanding of the mechanisms involved in outcompetition of organ tissue and colonization by primary and metastatic tumors. Unraveling and targeting molecular pathways that enhance competitive interactions among normal cells may aid in protecting tissues against initiation of neoplastic transformation. Due to the complex roles of cell competition in colorectal cancer, as well as the effects of mutational load, most findings are challenging to translate into the clinic. More research is required before clinical applications are an option. There is, however, one ongoing clinical trial based on two cell competition studies. As discussed earlier, APC mutant colorectal cancer cells secrete WNT antagonists such as NOTUM, which repress wild-type ISCs [119, 120]. As a consequence, these early transformed cells gain a competitive advantage that leads to their enhanced intestinal crypt occupancy and subsequent development into adenomas. However, cancer cell expansion can be prevented by lithium chloride administration. Lithium activates downstream WNT signaling in wild-type ISCs, which makes them insensitive to inhibition through NOTUM and other WNT antagonists. Currently, a clinical phase II trial is ongoing that studies the effects of lithium on Familial Adenomatous Polyposis patients [220]. This trial is of particular interest, as instead of decreasing the cellular fitness of cancer cells, which is the aim of most conventional therapies, the cellular fitness of wild-type cells is increased in this strategy. A drawback is that, as the treatment is chemo-preventive, it will primarily be beneficial for high-risk patients. However, the outcomes of this trial will be of great importance to FAP patients, as well as to the cancer field. How to decrease the cellular fitness of cancer cells relative to wild-type cells will remain an open question. Conversely, further understanding the tumor-promoting role of cell competition opens opportunities for targeted therapies aimed at disrupting or manipulating competitive interactions. Precision medicine approaches that selectively inhibit key mediators of cell competition hold promise for impeding cancer progression, providing a promising direction for anti-tumor and anti-metastatic interventions.
